# Non-Alcoholic Fatty Liver Disease Induced by Feeding Medium-Chain Fatty Acids Upregulates Cholesterol and Lipid Homeostatic Genes in Skeletal Muscle of Neonatal Pigs

**DOI:** 10.3390/metabo14070384

**Published:** 2024-07-11

**Authors:** Samuel D. Gerrard, Fernando H. Biase, Joseph A. Yonke, Ravi Yadav, Anthony J. Shafron, Nishanth E. Sunny, David E. Gerrard, Samer W. El-Kadi

**Affiliations:** 1 School of Animal Sciences, Virginia Tech, Blacksburg, VA 24601, USA; samuel15@vt.edu (S.D.G.); fbiase@vt.edu (F.H.B.); jayonke@vt.edu (J.A.Y.); yadavravi@vt.edu (R.Y.); ashafron23@vt.edu (A.J.S.); dgerrard@vt.edu (D.E.G.); 2 Department of Animal and Avian Sciences, University of Maryland, College Park, MD 20742, USA; nsunny@umd.edu

**Keywords:** gene expression, skeletal muscle, metabolism, NAFLD, neonatal pig

## Abstract

Non-alcoholic fatty liver disease (NAFLD) is a range of disorders characterized by lipid accumulation in hepatocytes. Although this spectrum of disorders is associated with adult obesity, recent evidence suggests that this condition could also occur independently of obesity, even in children. Previously, we reported that pigs fed a formula containing medium-chain fatty acids (MCFAs) developed hepatic steatosis and weighed less than those fed an isocaloric formula containing long-chain fatty acids (LCFAs). Our objective was to determine the association between NAFLD and the skeletal muscle transcriptome in response to energy and lipid intake. Neonatal pigs were fed one of three formulas: a control formula (CONT, *n* = 6) or one of two isocaloric high-energy formulas containing either long (LCFA, *n* = 6) or medium (MCFA, *n* = 6) chain fatty acids. Pigs were fed for 22 d, and tissues were collected. Body weight at 20 and 22 d was greater for LCFA-fed pigs than their CONT or MCFA counterparts (*p* < 0.005). Longissimus dorsi weight was greater for LCFA compared with MCFA, while CONT was intermediate (*p* < 0.05). Lean gain and protein deposition were greater for LCFA than for CONT and MCFA groups (*p* < 0.01). Transcriptomic analysis revealed 36 differentially expressed genes (DEGs) between MCFA and LCFA, 53 DEGs between MCFA and CONT, and 52 DEGs between LCFA and CONT (FDR < 0.2). Feeding formula high in MCFAs resulted in lower body and muscle weights. Transcriptomics data suggest that the reduction in growth was associated with a disruption in cholesterol metabolism in skeletal muscles.

## 1. Introduction

Non-alcoholic fatty liver disease (NAFLD) is a spectrum of progressive hepatic disorders that can range from simple hepatic steatosis to hepatocellular carcinoma [1,2]. While obesity is one of the largest risk factors [3], recent data suggest that the prevalence of this disorder may be greater than previously thought in younger individuals [4]. Nutritional factors such as high dietary fat [5] and carbohydrates [6] are most commonly associated with the onset of NAFLD. In this context, we previously reported that, compared with long-chain fatty acids (LCFA), feeding a formula rich in medium-chain fatty acids (MCFA) resulted in panacinar hepatic steatosis in neonatal pigs [7,8], which is the prevalent form in pediatric patients [9,10]. Moreover, panacinar hepatic steatosis coincided with a reduction in growth [7]. It was not clear from our previous studies whether the reduction in growth was correlated with changes in fat metabolism in skeletal muscles.

The neonatal period is marked by faster growth rates than any other postnatal stage, primarily driven by skeletal muscle hypertrophy and protein deposition [11]. In this connection, since skeletal muscles make up approximately 25% and 30–40% of total body mass in neonates [12] and adults [13], they play a crucial role in maintaining energy homeostasis [14]. While disruption of fatty acid metabolism in the liver and its association with NAFLD have been extensively studied [15], less is known about the relationship of this disease with skeletal muscle metabolism. It is now recognized that disruption of metabolic processes of the adipose–muscle–liver axis may exacerbate the disease [16,17]. Moreover, disruption of the metabolic capacity of muscles would be further aggravated as a result of slow growth rates and may play a role in worsening chronic metabolic disorders. The extent to which energy homeostasis between the liver and peripheral tissues is disrupted has yet to be elucidated in neonates. We previously reported that feeding formulas rich in medium-chain fatty acids caused severe hepatic steatosis with a concomitant reduction in skeletal muscle growth in neonatal pigs [7]. We set out to examine whether changes in the expression of genes related to lipid metabolism in muscles would coincide with hepatic steatosis. Our hypothesis was that feeding a high-fat MCFA formula alters the transcriptome of skeletal muscles. Our objectives were to: (1) identify differentially expressed genes in the skeletal muscles of pigs in response to excess energy intake; and (2) compare transcript abundance in pigs fed MCFA with those fed LCFA formulas to understand the impact of NAFLD on skeletal muscle lipid metabolism.

## 2. Methods

### 2.1. Animals and Diets

All animal protocols were approved by the Virginia Tech Animal Care and Use Committee. Briefly, eighteen 3-day-old Duroc × Landrace × Yorkshire pigs were separated from the sow and fed the control (CONT) formula (Table 1). Animals were housed in temperature-controlled rooms kept at 30 °C with a 12 h light–dark cycle. The pigs used in the current study comprised a smaller cohort compared with the larger group of pigs we previously used [7]. After a 4 d acclimatization to their environment, pigs were allocated to experimental formulas: a low-energy CONT formula (*n* = 6), high-energy formulas using long-chain fatty acids (LCFAs, *n* = 6), or an isocaloric high-energy formula using medium-chain fatty acids (MCFA, *n* = 6). Body weights were measured on a digital scale (Ohaus, Parsippany, NJ, USA) every other day for the duration of the study to monitor growth and adjust the formula amount, which was set at 250 mL · kg body weight^−1^·d^−1^ divided into 5 equal meals. Pigs were fed for 22 days and then euthanized for sample collection and organ weights.

### 2.2. Dual-Energy X-ray Absorptiometry

Body composition was measured using dual-energy X-ray absorptiometry in the small animal mode (DXA, GE Healthcare, Chicago, IL, USA) as previously described [7]. Measurements were taken on days 1 and 21 and adjusted for the start and sampling dates according to weight. Raw measurements were used in the calculation of lean gain, protein efficiency, energy deposited, energy efficiency, protein deposition, and fat accretion.

### 2.3. RNA Extraction

Frozen longissimus dorsi tissue was ground with a mortar and pestle, and 50 mg was weighed into a 2 mL Eppendorf tube. A commercially available kit (Direct-zol RNA Miniprep Kit, Zymo Research, Orange, CA, USA) was used to extract RNA. Briefly, chloroform was added to all samples and mixed vigorously. Samples were then centrifuged at 12,000× *g* for 15 min at 4 °C. The aqueous phase was transferred to a new tube, and one volume of 100% ethanol was added to the sample. The RNA was then bound onto the Zymo-Spin^TM^ IIC column and washed according to the manufacturer’s instructions. Purity and concentration were measured using a spectrophotometer (NanoDrop, Thermo Fisher Scientific, Wilmington, DE, USA) as previously described [18]. Samples were sent to Novogene (Sacramento, CA, USA) for RNA library preparation, quality control, and sequencing to produce pair-end sequences 150 nucleotides long.

### 2.4. RNA Sequencing

Adapters were removed from the sequences using trimmomatic (v 0.39) [19], retaining only sequences with 100 or more nucleotides in length and an average quality of 30 or greater. Then, reads were aligned to the pig reference genome (Sus_scrofa.Sscrofa11.1.dna.toplevel.fa) downloaded from Ensembl [20,21] and the database (https://www.ncbi.nlm.nih.gov/datasets/genome/GCF_000003025.6/, accessed on 4 October 2023) with hisat2 (v 2.2.1) [22] using the very sensitive parameter. Next, Samtools (v 1.10) [23] was used to retain reads with one match on the genome, followed by the removal of duplicates using the bammarkduplicates function in biobambam2 (v 2.0.95) (https://github.com/gt1/biobambam/tags, accessed on 4 October 2023) as previously described [24]. Lastly, only sequences with more than 90% correct alignment were used for counting.

### 2.5. Data Analysis and Transcriptomics

Treatments were assigned to experimental units using a complete randomized design. Data for continuous variables were analyzed by ANOVA of the MIXED procedure of SAS (version 9.4, SAS Institute Inc., Cary, NC, USA). When significance was detected, means were compared using the Turkey–Kramer Multiple Comparison Test. Data were reported as means ± SE, and differences were considered significant when *p* < 0.05. The number of fragments matched to Ensembl [20,21] and pig gene annotation (Sus_scrofa.Sscrofa11.1.111) [24] were counted using featureCounts [25]. Counts were loaded into R software (Version 4.4.0) [26]. We preserved genes annotated as protein-coding, pseudogenes, or long non-coding RNA. Genes were retained for further analysis if counts per million (CPM) > 4 and fragments per kilobase per million (FPKM) > 1 in at least four samples. We compared transcript abundance between samples from each group using the R packages ‘edgeR’ [27,28], with the quasi-likelihood test, and ‘DEseq2’ [29], using Wald’s and likelihood tests. Tests considered treatment and sex as fixed effects in the model. The nominal *p*-values of both tests were corrected for multiple hypothesis testing using the false discovery rate (FDR) method [30]. Differential transcript abundance was assumed when FDR < 0.2 for both tests. We carried out tests for enrichment or gene ontology categories using the “goseq” package [31] in R software. In all tests, genes whose transcript abundances were estimated for the samples being tested were used as the background. The nominal *p*-value for multiple hypothesis testing was adjusted by controlling the familywise error rate (FWER) following the previously reported method [32] using the function “p.adjust” from the ‘stats’ R package. We inferred significance when FWER < 0.2. 

## 3. Results

### 3.1. Growth Performance and Organ Weights

**Figure 1:**
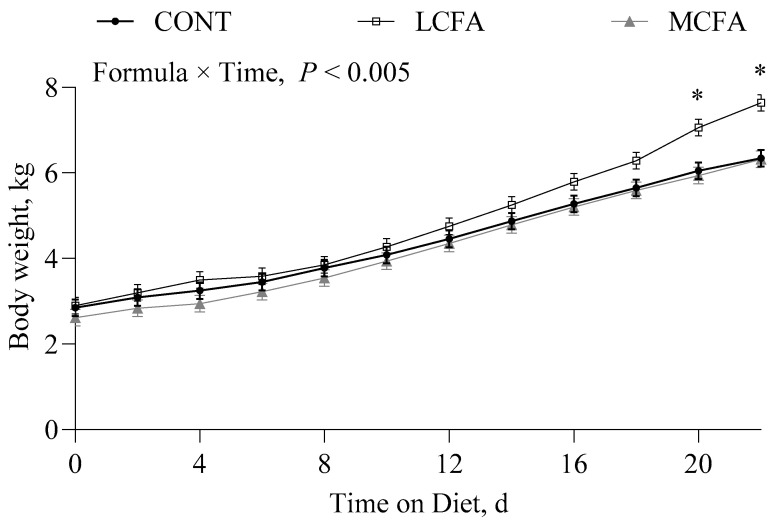
Body weight of neonatal pigs fed either a low-energy control formula (CONT), isocaloric high-energy formulas rich in long-chain (LCFA), or medium-chain (MCFA) fatty acids for 22 d. Statistical analyses were completed using mixed-model ANOVA. Values are means ± SEM; *n* = 5–6/treatment. When a significant effect was detected, means were compared using the Tukey–Kramer post hoc test. * Indicates that the means of LCFA pigs differed from those of CONT and MCFA pigs, *p* ≤ 0.05.

**Figure 2:**
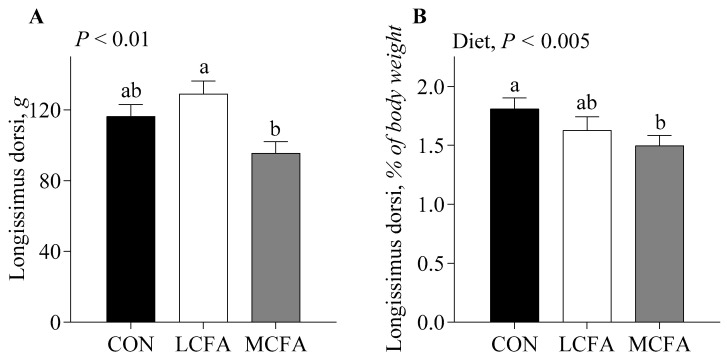
Longissimus dorsi weight (**A**) and weight as a percentage of body weight (**B**) of neonatal pigs fed either a low-energy control formula (CONT) or isocaloric high-energy formulas rich in long-chain (LCFA) or medium-chain (MCFA) fatty acids for 22 d. Statistical analyses were completed using a mixed-model ANOVA. Values are means ± SEM; *n* = 5–6/treatment. When a significant effect was detected, means were compared using the Tukey–Kramer post hoc test. ^a,b^ Means without a common letter differ, *p* ≤ 0.05.

**Figure 3:**
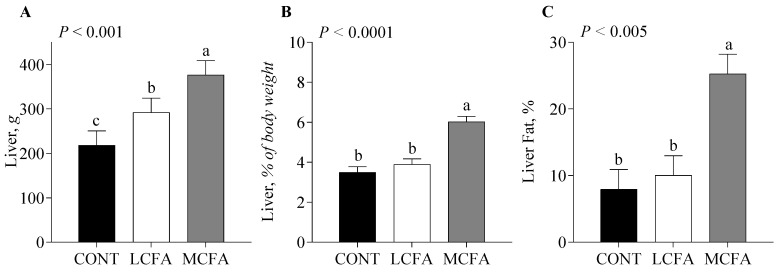
Liver weight (**A**), weight as a percentage of body weight (**B**), and fat as a percentage of liver weight (**C**) of neonatal pigs fed either a low-energy control formula (CONT) or isocaloric high-energy formulas rich in long-chain (LCFA) or medium-chain (MCFA) fatty acids for 22 d. Statistical analyses were completed using mixed-model ANOVA. Values are means ± SEM; *n* = 5–6/treatment. When a significant effect was detected, means were compared using the Tukey–Kramer post hoc test. ^a–c^ Means without a common letter differ, *p* ≤ 0.05.

Pigs fed the LCFA had greater body weights (Figure 1) on days 20 and 22 of the study compared with the CONT and MCFA groups (*p* < 0.05). Longissimus dorsi (Figure 2A) weight was greatest for pigs fed the LCFA compared with those fed the MCFA formula, while those fed the CONT formula were intermediate (*p* < 0.05). When expressed as a percentage of body weight, longissimus dorsi (Figure 2B) was greater for the CONT group compared with MCFA, while the LCFA group was intermediate (*p* < 0.005). Liver weight was greater (*p <* 0.001) for pigs in the MCFA group compared with those in the LCFA group, and the liver weight of pigs fed the LCFA formula was greater than those fed the CONT formula (Figure 3A). Liver weight as a percent of body weight (Figure 3B) and hepatic fat as a percentage of liver weight (Figure 3C) were greater for pigs fed the MCFA than those fed either LCFA or CONT formulas (*p <* 0.001; *p* < 0.005).

### 3.2. Energy Utilization

**Figure 4:**
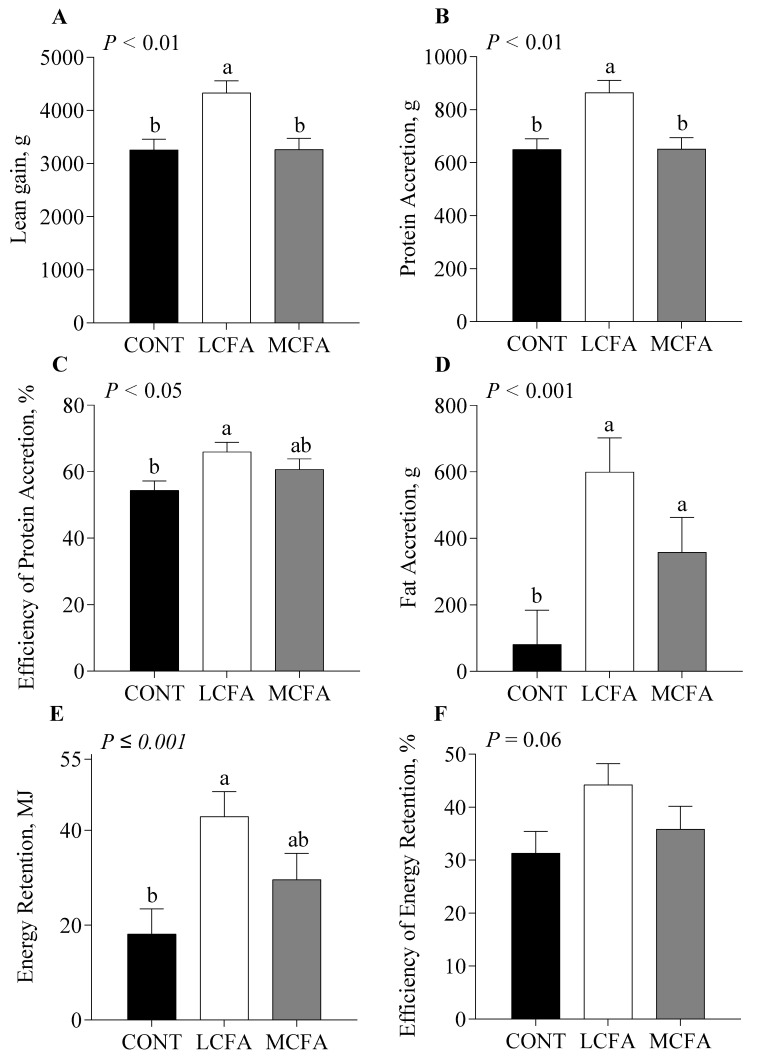
Efficiency of energy and protein retention of neonatal pigs fed either a control formula (CONT) or isocaloric high-energy formula with additional energy from long-chain fatty acids (LCFAs) or medium-chain fatty acids (MCFAs) for 22 days. Lean gain (**A**), protein accretion (**B**), efficiency of protein accretion (**C**), fat accretion (**D**), energy retention (**E**), and efficiency of energy rentention (**F**) were calculated from DXA measurements. Statistical analyses were completed using a mixed-model ANOVA. Values are means ± SE; *n* = 5–6/treatment. When a significant effect was detected, means were compared using the Tukey–Kramer posthoc test. ^a,b^ Means without a common letter differ, *p* ≤ 0.05.

Lean gain (Figure 4A) was greater for pigs in the LCFA group compared with those in the CONT and MCFA groups (*p* < 0.01). Protein accretion (Figure 4B) was greater for pigs fed LCFA compared with those fed CONT and MCFA formulas (*p* < 0.010). The efficiency of protein accretion (Figure 4C) was greatest for pigs in the LCFA group, least for pigs in the CONT group, and intermediate for those in the MCFA group (*p* < 0.05). Fat accretion (Figure 4D) was less for pigs fed the CONT formula compared with those fed either LCFA or MCFA formulas (*p* < 0.001). However, energy retention (Figure 4E) was greatest for pigs in the LCFA group, least for pigs in the CONT group, and intermediate for pigs in the MCFA group (*p ≤* 0.001). In contrast, the efficiency of energy retention (Figure 4F) was not different among treatments.

### 3.3. Transcriptomics

**Figure 5:**
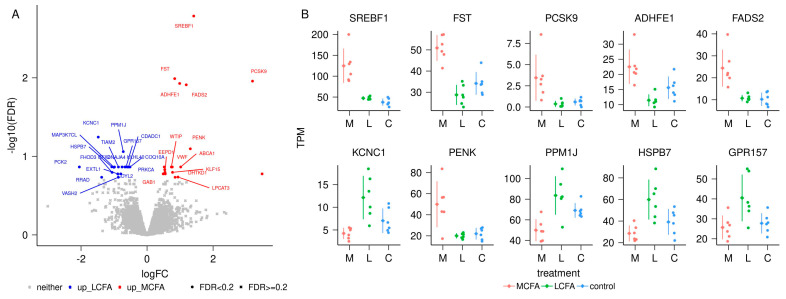
Differential transcript abundance between medium-chain fatty acids (M) and long-chain fatty acids (L). (**A**) Volcano plot of the contrast M versus L. (**B**) Data points of the top 10 genes with differential transcript abundance in transcripts per million (TPM) for the contrast M versus L. In each category, the dots represent data points for individual pigs, the diamond to the left of the dots indicates the average, and the vertical bars indicate the standard deviation.

**Figure 6:**
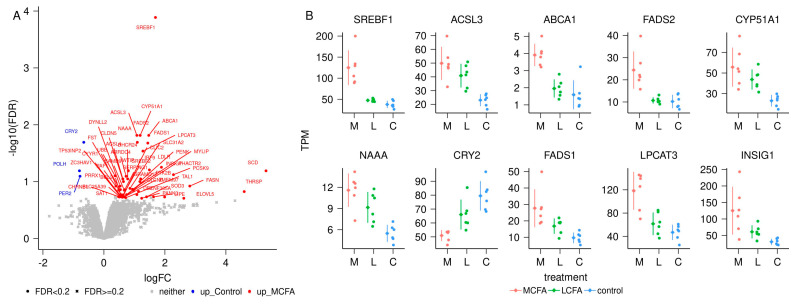
Differential transcript abundance between medium-chain fatty acids (M) and control (C) samples. (**A**) Volcano plot of the contrast M versus C. (**B**) Data points of the top 10 genes with differential transcript abundance in transcripts per million (TPM) for the contrast M versus C. In each category, the dots represent data points for individual pigs, the diamond to the left of the dots indicates the average, and the vertical bars indicate the standard deviation.

**Figure 7:**
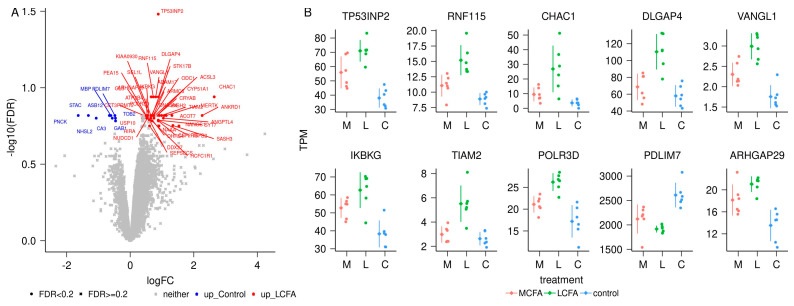
Differential transcript abundance between long-chain fatty acids (L) and control (C) samples. (**A**) Volcano plot of the contrast L versus C. (**B**) Data points of the top 10 genes with differential transcript abundance in transcripts per million (TPM) for the contrast L versus C. In each category, the dots represent data points for individual pigs, the diamond to the left of the dots indicates the average, and the vertical bars indicate the standard deviation.

**Figure 8:**
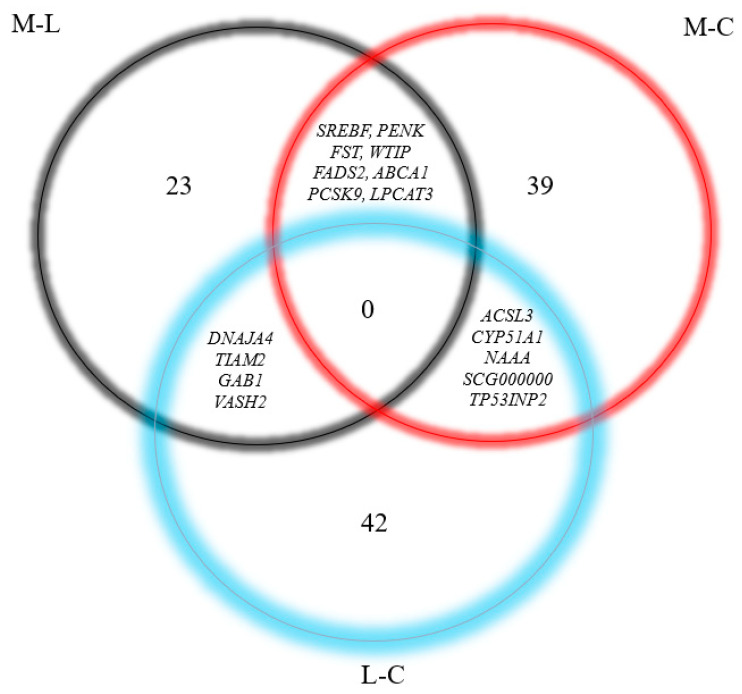
Venn diagrams representing DEGs in each contrast from neonatal pigs fed either a control formula (C) or isocaloric high-energy formulas with additional energy from long-chain fatty acids (L) or medium-chain fatty acids (M) for 22 days. Overlapping gene symbols are listed between contrasts.

Overall, we produced 259,672,636 pairs of reads that yielded an average of 7,461,274 ± 1,600,980 pairs of reads per sample, mapped to the annotation and being used for transcriptome analysis. Altogether, 11,285 genes (10,974 protein-coding, 288 lncRNA, and 23 pseudogenes) were analyzed. There were 36 differentially expressed genes (DEG) in the MCFA vs. LCFA (M-L) contrast (Table S1 and Figure 5) with an FDR < 0.2. The contrast between MCFA and CONT (M-C) identified 53 DEG (Table S2 and Figure 6). Similarly, the contrast between LCFA and CONT (L-C) identified 52 DEG (Table S3 and Figure 7). Contrasts M-L and M-C identified eight genes that were differentially expressed in the MCFA when compared with both LCFA and CONT (Figure 8). Notably, the DEGs from the M-C contrast were enriched for biological processes important for NAFLD, such as “cholesterol homeostasis” (*ABCA1*, *GRAMD1B*, *INSIG1*, *LDLR*, *MYLIP*, *PCSK9*, *SREBF2*, *TMEM97*) and “lipid metabolic process” (*ACSL3*, *ACSL4*, *FADS1*, *FADS2*, *INSIG1*, *LDLR*, *LIPE*, *NAAA*, and *SCD*; Table 2 and Table S4).

## 4. Discussion

NAFLD is closely associated with obesity and metabolic syndrome through metabolic disruptions and altered fat metabolism in several organs [33]. This disease not only has an impact on liver health and longevity but also influences peripheral tissues such as skeletal muscle, thereby negatively affecting the growth of neonates and adolescents [34]. Previously, we reported that feeding neonatal pigs a high-fat formula containing MCFA leads to greater fat accumulation in the liver and lesser body weights compared with those fed an isocaloric LCFA formula [7,8]. Skeletal muscles make up a significant portion of body weight in neonates [12], suggesting that the body weight difference between LCFA and MCFA pigs might be attributed to variations in muscle mass. In addition to hepatic fat accumulation and lower body weights, we have determined that high-fat MCFA formulas upregulate fatty acid metabolic genes in the liver, contributing to hepatic steatosis. Moreover, the liver plays a key role in fatty acid packaging and energy distribution throughout the body [35]. The involvement of fatty acid metabolism in skeletal muscle and its impact on the development of NAFLD remain to be determined. Therefore, we set out to test the hypothesis that feeding a high-fat MCFA formula alters the transcriptome of skeletal muscles to increase lipid metabolism. Our objectives were to identify differentially expressed genes in the skeletal muscles of pigs in response to excess energy intake and to compare transcript abundance in pigs fed MCFA compared with those fed LCFA formulas to understand the impact of NAFLD on skeletal muscle metabolism.

Analysis of biological pathways indicated that the MCFA group was different from CONT; however, this may be due to greater dietary energy in MCFA compared with those fed the CONT formula. Therefore, exploring DEGs in the M versus L contrast would be more appropriate to underscore the effects of those fat sources as opposed to high vs. low energy. To that end, sterol regulatory element binding factor (*SREBF1*) was upregulated in MCFA pigs when compared between the LCFA and CONT groups. This gene encodes for SREBP-1, which can be posttranscriptionally modified into two isoforms, 1a and 1c, that both have an impact on lipid and cholesterol metabolism [36]. Although, mechanistically, the upregulation in SREBP-1 cannot be explained by the current data, we speculate that MCFA could alter non-esterified fatty acids (NEFAs) in plasma. As such, it was reported that circulating NEFAs are greater in NAFLD patients compared with healthy individuals [37]. Thus, it is likely that NEFAs would cause an upregulation of SREBP and enhance the upregulation of genes related to fat metabolism [38]. Overexpression of the SREBP-1 protein also reduces the expression of muscle growth-related genes and decreases markers of differentiation in vivo and in vitro [39]. Moreover, overexpression of SREBP-1 reduces muscle protein synthesis with a lesser reduction in protein degradation, thus suppressing hypertrophy [36]. Herein, it is likely that MCFA feeding may suppress lean tissue accretion via upregulation of *SREBF1* in skeletal muscles compared with LCFA. If this is indeed pathological, there may be compensatory mechanisms by which MCFA-fed pigs dampen the effects of SREBP-1 overexpression since these pigs grew throughout the study, albeit to a lesser extent than LCFA-fed pigs.

Regulation of SREBP-1 through the insulin-induced gene (INSIG1) further indicated upregulation of *SREBF1.* INSIG1 inhibits SREBP-1 by binding the SCAP complex, keeping it localized on the endoplasmic reticulum membrane [40] and preventing its maturation and entry to the nucleus [41]. Herein, INSIG1 was upregulated in the MCFA compared with the CONT group; however, the magnitude of INSIG1 expression was less than that of SREBP-1, suggesting a dampened inhibitory effect on SREBP-1. It remains to be elucidated whether greater INSIG1 expression would be enough to suppress the SREBP-1 protein.

It is worth noting that there are two key differences between the fat sources that were used in the study. In addition to chain-length differences between MCFA and LCFA, the degree of saturation of these fatty acids is also different (MCFA 92% and LCFA 41%). In the current study, fatty acid desaturase 2 (*FADS2*) was upregulated in MCFA compared with the LCFA and CONT groups. Although there is a scarcity of studies investigating the effect of differential expression of FADS1/2 in skeletal muscle, reports suggest that feeding highly saturated fatty acids alone does not alter hepatic *FADS1/2* expression in mice [42]. Herein, upregulation of *FADS2* in the skeletal muscle of pigs fed the MCFA compared with those fed the LCFA suggests that those effects may be either due to differences in chain lengths or to differences in the saturated fatty acid content of the formulas. While this is a plausible explanation, further studies are needed to determine whether this is the cause of the upregulation of these genes.

Lipid and cholesterol metabolic pathways intersect via lipoproteins such as low-density lipoprotein (LDL), which carry fatty acids and cholesterol throughout the body [43]. LDL is trafficked into cells through the LDL receptor (LDLR), and the expression of *LDLR* is controlled by SREBP-1 [44]. Therefore, NEFAs can increase the transcription of LDLR through the activation of SREBP, which aligns with the current data. Moreover, myosin regulatory light chain interacting protein (*MYLIP*) was upregulated in MCFA pigs, highlighting the downstream metabolism of cholesterol once it enters the cell. This ubiquitin ligase plays a role in the degradation of LDLR, very low-density lipoprotein receptor (VLDLR), and low-density lipoprotein receptor-related protein 8 (LRP8). While these genes are only upregulated in MCFA-fed pigs compared with CONT in the current study, regulation of LDLR receptors is upregulated in MCFA compared with both LCFA and CONT-fed pigs. This is through proprotein convertase subtilisin/kexin type 9 (*PCSK9*), which aids in the degradation of LDLR [45]. Treatment of hypercholesterolemia with statins counteracts the disease in part through upregulation of *PCSK9* [46]. Upregulation of *PCSK9* in the MCFA group is likely to reduce cholesterol entry into muscles. Circulating cholesterol is high in coconut oil-fed pigs, as it significantly increases LDL compared with other oils [47]. Moreover, it is also likely that MCFA pigs have more circulating cholesterol moving into skeletal muscles. Upregulation of ATP binding cassette subfamily A member 1 (*ABCA1*) in MCFA pigs compared with LCFA and CONT supports this view.

ABCA1 is vital for reverse cholesterol transport because it packages phospholipid and cholesterol onto apo-A1 to form high-density lipoprotein (HDL). This protein is found in skeletal muscle and is highly correlated with mRNA expression [48]. While a significant amount of cholesterol is synthesized in the liver, cholesterol could also be synthesized in skeletal muscles [49]. Herein, it appears that skeletal muscles may also contribute to cholesterol synthesis despite the lack of differences in the expression of HMG-CoA reductase, the rate-limiting enzyme of cholesterol synthesis. In the current study, endonuclease/exonuclease/phosphatase family domain containing 1 (*EEPD1*) was upregulated in pigs fed the MCFA compared with those fed the LCFA but not those fed the CONT formula. This gene is a proposed target of the liver X receptor (LXR) that can aid in cholesterol movement across the cell membrane [50]. In fact, LXR has also been reported to play a role in regulating cholesterol efflux from skeletal muscles [51]. While the mechanism for the upregulation of this gene is not clear from this study, it highlights a transcriptomic difference between the skeletal muscles of pigs fed MCFA and LCFA. Taken together, these data are in support of previous studies suggesting that skeletal muscle plays a role in cholesterol homeostasis [51] and that MCFA feeding augments this role. Thus, there are two likely explanations for the observed differences. The first is that disruption in cholesterol homeostasis occurs first in the liver, during the development of NAFLD, and evolves in skeletal muscle, where it exacerbates the pathogenesis. However, it cannot be ruled out that disruption in cholesterol homeostasis in skeletal muscles preceded hepatic dysregulation. Further studies are needed to elucidate this process.

The ability of a cell to keep charge across the plasma membrane is imperative to its survival. Electrochemical gradients are maintained through ion channels and pumps that use ATP to move ions against a gradient. Data from the current study suggest that the monoatomic ion transport pathway was upregulated in MCFA compared with CONT pigs, specifically two chloride transports, *CLIC2* and *CLCN6*, a copper transporter, and an acetylcholine channel. Changes in ion transport are well studied as a product of inflammatory disease [52]. In fact, high-fat diets could cause inflammation due to an abundance of fat accumulation in tissues [1,53]. In the current study, there was a reduction in potassium and calcium transporters in MCFA compared with LCFA-fed pigs. A reduction in cellular uptake of potassium can ensue during potassium deprivation to increase levels of extracellular potassium [54]. While the exact cause of the reduction in ion transporters is not clear, disruption of calcium transport may be related to cholesterol metabolism. High levels of membrane cholesterol cause an increase in cytosolic calcium ions through increased membrane permeability [55]. We speculated that the downregulation of calcium transporters is likely a compensatory mechanism to inhibit calcium ion entry into cells. While this is a plausible explanation, more studies are needed to show if this is the correct mechanism of action.

## 5. Conclusions

Neonatal pigs afflicted with NAFLD as a result of high-fat formulas containing MCFAs were unable to attain weights comparable to those fed an LCFA source. Our data suggest that the low-fat CONT and high-fat MCFA gained less lean mass compared with those fed the high-fat LCFA formula. Our data suggest that pigs fed MCFA developed NAFLD and had greater expression of cholesterol and lipid metabolic genes, including lipoprotein trafficking. This occurred concomitantly with changes in the expression of genes related to ion movement and membrane integrity. Our data indicated that the differential expression of genes related to cholesterol and fatty acid metabolism was associated with NAFLD in skeletal muscles. Whether changes in muscle metabolism precede or result from the development of NAFLD remains to be determined.

## Figures and Tables

**Table 1:** Ingredients and nutrient composition of a low-energy control formula (CONT) or high-energy isocaloric formulas rich in long-chain (LCFA) or medium-chain (MCFA) fatty acids fed to pigs for 22 d.

	CONT	LCFA	MCFA
Ingredients, %			
Water	86	82	82
Whey protein isolate ^1^	5.6	5.6	5.6
Fat Pak 80 ^1,^*	5.4	10.1	-
Coconut oil	-	-	8.8
Lactose **	0.5	-	1
Trace mineral premix ^2,‡^	0.9	0.9	0.9
Dicalcium phosphate	0.5	0.5	0.6
Casein	0.2	-	0.5
Calcium carbonate	0.2	0.2	0.2
Vitamin premix ^2,§^	0.2	0.2	0.2
Xanthum gum	0.1	0.1	0.1
Calculated Analysis			
Protein, %	5.3	5.3	5.3
Fat, %	4.4	8.2	8.8
Lactose, %	1.0	1.0	1.0
Calcium, %	0.23	0.23	0.23
Phosphorus, %	0.14	0.14	0.14
Metabolizable Energy, Kcal/kg	656	984	984

^1^ Milk Specialties, Eden Prairie, MN. ^2^ Dyets Inc., Bethlehem, PA. * Contains: crude protein, 4%; crude fat, 80%; lactose, 9.6%; 8150 kcal/kg. ** Added lactose to bring the formula total to 1%. ^‡^ Trace mineral premix provided (g/kg): calcium phosphate, dibasic, 187; calcium carbonate, 279; sodium chloride, 85; potassium phosphate, monobasic, 155; magnesium sulfate, anhydrous, 44; manganous carbonate, 0.93; ferric citrate, 10; zinc carbonate, 1.84; cupric carbonate, 0.193; potassium iodate, 0.005; sodium selenite, 0.007. ^§^ Vitamin premix provided (g/kg): thiamine HCl, 0.1; riboflavin, 0.375; pyridoxine HCl, 0.1; niacin, 1; calcium pantothenate, 1.2; folic acid, 0.13; biotin, 0.02; cobalamin, 1.5; retinyl palmitate, 0.8; cholecalciferol, 0.05; tocopheryl acetate, 8.8; menadione sodium bisulfate, 0.08.

**Table 2:** Biological pathway enrichment in a medium-chain fatty acid group compared with the control group (M-C) ^1^.

Contrast	GO BP ID	Term	N Genes	Enrichment	FWER
M-C	GO:0042632	Cholesterol homeostasis	8	41.5	1.8 × 10^−10^
M-C	GO:0008203	Cholesterol metabolic process	6	31.9	4.5 × 10^−7^
M-C	GO:0006629	Lipid metabolic process	9	11.3	1.5 × 10^−6^
M-C	GO:0006631	Fatty acid metabolic process	4	14.9	0.0014
M-C	GO:0006811	Monoatomic ion transport	4	4.7	0.080
M-C	GO:0000122	Negative regulation of transcription by RNA polymerase II	6	20.6	0.1884

^1^ Contrast—comparison between CONT (C), MCFA (M), or LCFA (L); GO BP ID—gene ontology biological process identification; term—name of this pathway; N genes—number of genes in this pathway; enrichment—fold change in the outlined term; FWER—family-wise error rate.

## Data Availability

Dataset available on request from the authors.

## Supplementary Materials

The following supporting information can be downloaded at: https://www.mdpi.com/article/10.3390/metabo14070384/s1, Table S1: Analysis of differential transcript abundance between medium (M) versus long (L) -chain fatty acids samples.; Table S2: Analysis of differential transcript abundance between medium-chain fatty acids (M) versus control (C) samples.; Table S3: Analysis of differential transcript abundance between long-chain fatty acids (L) versus control (C) samples.; Table S4: Analysis of biological process of differentially expressed genes in the contrast between medium-chain fatty acids (M) versus control (C) samples.

## Abbreviations

Non-alcoholic fatty liver disease (NAFLD), non-alcoholic steatohepatitis (NASH), medium-chain fatty acid (MCFA), long-chain fatty acid (LCFA), dual-energy X-ray absorptiometry (DEXA), longissimus dorsi (LD), sterol regulatory element binding factor (*SREBF1*), non-esterified fatty acids (NEFAs), fatty acid desaturase 2 (*FADS2*), low-density lipoprotein (LDL), LDL is brought into cells through LDL receptor (LDLR), myosin regulatory light chain interacting protein (*MYLIP*), very low-density lipoprotein (VLDL), low-density lipoprotein receptor-related protein 8 (LRP8), low-density lipoprotein receptor-related protein 8 (LRP8), ATP binding cassette subfamily A member 1 (*ABCA1*), high-density lipoprotein (HDL), dehydrocholesterol reductase (*DHCR24*), endonuclease/exonuclease/phosphatase family domain containing 1 (*EEPD1*), liver x receptor (LXR).
